# A Re-Emerging Marker for Prognosis in Hepatocellular Carcinoma: The Add-Value of FISHing c-myc Gene for Early Relapse

**DOI:** 10.1371/journal.pone.0068203

**Published:** 2013-07-10

**Authors:** Federica Pedica, Andrea Ruzzenente, Fabio Bagante, Paola Capelli, Ivana Cataldo, Serena Pedron, Calogero Iacono, Marco Chilosi, Aldo Scarpa, Matteo Brunelli, Anna Tomezzoli, Guido Martignoni, Alfredo Guglielmi

**Affiliations:** 1 Azienda Ospedaliera Universitaria Integrata di Verona, FISH Molecular Laboratory, Department of Pathology and Diagnostic, University of Verona, Verona, Italy; 2 Department of Surgery, Division of General Surgery "A", GB Rossi Hospital, University of Verona Medical School, University of Verona, Verona, Italy; 3 Azienda Ospedaliera Universitaria Integrata di Verona, dO DAI Pathology and Diagnostic, Verona, Italy; The Chinese University of Hong Kong, Hong Kong

## Abstract

Hepatocellular carcinoma is one leading cause of cancer-related death and surgical resection is still one of the major curative therapies. Recently, there has been a major effort to find mechanisms involved in carcinogenesis and early relapse. c-myc gene abnormality is found in hepatocarcinogenesis. Our aim was to analyze the role of c-myc as prognostic factor in terms of overall survival and disease-free survival and to investigate if c-myc may be an important target for therapy. We studied sixty-five hepatocellular carcinomas submitted to surgical resection with curative intent. Size, macro-microvascular invasion, necrosis, number of nodules, grading and serum alfa-fetoprotein level were registered for all cases. We evaluated the c-myc aberrations by using break-apart FISH probes. Probes specific for the centromeric part of chromosome 8 and for the locus specific c-myc gene (8q24) were used to assess disomy, gains of chromosomes (polysomy due to polyploidy) and amplification. c-myc gene amplification was scored as 8q24/CEP8 > 2. Statistical analysis for disease-free survival and overall survival were performed. At molecular level, c-myc was amplified in 19% of hepatocellular carcinoma, whereas showed gains in 55% and set wild in 26% of cases. The 1- and 3-year disease-free survival and overall survival for disomic, polysomic and amplified groups were significantly different (p=0.020 and p=.018 respectively). Multivariate analysis verified that the AFP and c-myc status (amplified vs. not amplified) were significant prognostic factors for overall patients survival. c-myc gene amplification is significantly correlated with disease-free survival and overall survival in patients with hepatocellular carcinoma after surgical resection and this model identifies patients with risk of early relapse (≤12 months). We suggest that c-myc assessment may be introduced in the clinical practice for improving prognostication (high and low risk of relapse) routinely and may have be proposed as biomarker of efficacy to anti-c-myc targeted drugs in clinical trials.

## Introduction

Hepatocellular carcinoma (HCC) is the sixth most common neoplasm worldwide and third leading cause of cancer-related mortality, responsible for 600,000 deaths annually [1]. Surgery is currently one of the curative treatments for HCC and 5-year overall survival after liver resection can reach 40-70% of patients [2]. Actually, recurrence is still the major issue after surgery and an important prognostic factor in term of OS [3,4]. Moreover early relapses (< 12 months) are frequent, almost fifty per cent of cases, and are significantly related to short survival after resection, lower than 40% at 3-year for patients with early recurrence [5,6].

In recent years, there has been a major efforts to identify the key cellular mechanisms involved in early recurrence and poor prognosis after resection of HCC [7]. At molecular level, no definitive tissue biomarkers have been actually encountered in the management of patients, routinely. c-myc inhibitors and drugs with efficacy to molecular mechanisms involved in the c-myc pathways are emerging in different oncological contexts [8,9].

Fluorescence in situ hybridization (FISH) technical analysis offers one of the most sensitive, specific, and reliable strategies (i.e the Her-2 status in breast [10] and gastric carcinoma [11], ALK gene in lung carcinoma [12], 1p and 19q chromosomal status in oligodendroglioma [13], for identifying acquired chromosomal changes on routinely available formalin-fixed and paraffin embedded tissue sections.

c-myc gene is implicated in a great number of cancers because it coordinates the upregulation of a transcriptional program for cell division, cell metabolism and survival [14]. Recently the problem of targeting c-myc for cancer treatment has been pursued [15]. Commercially robust probes for FISH analysis on formalin fixed tissue samples are actually available.

In the present study we sought to evaluate the molecular status of c-myc by using the FISH method to assess the importance of c-myc in HCC carcinogenesis and its prognostic significance in terms of disease free survival (DFS) and overall survival (OS) in a cohort of patients submitted to liver resection with curative intent for HCC.

## Materials and Methods

### Ethic Statements

We used tissue samples from human participants. All tissue blocks have been previously declaired to be available for the purposes of the actual study by the Istitutional Review Board (study conducted according to the principles expressed in the Declaration of Helsinki).

Our institutional review board and the ethics committee approved the original human work that produced the tissue samples (Azienda Ospedaliera Integrata di, Verona, Verona, Italy and Director of the Department of Pathology and Diagnostic).

All processing in obtaining the material has been performed after a written informed consent.

### Case selection

From January 2006 to December 2009, 104 liver resections with curative intent for HCC were performed in a single Division of Surgery of the Department of Surgery (University of Verona, Italy). Among these, the last 65 consecutive patients were analyzed by FISH and were subjects of this study.

Before liver resection, all patients had serum liver function tests (bilirubin, alkaline phosphatase, transaminase, albumin, prothrombin time), viral hepatitis tests (HBsAg, HBsAb, HBc tot. Ab, anti-HCV Ab), blood count, serum creatinine level, chest radiography, liver ultrasonography and abdominal-CT or abdominal MRI.

After resection all patients underwent regular follow up with serum alpha-fetoprotein level (AFP) dosage and ultrasonography every 6 months. Suspect recurrences were confirmed with computed tomography (CT) or magnetic resonance imaging (MRI). Chest CT or bone scan were performed in case of recurrence or clinical suspect of distant metastases.

All recurrences were evaluated for new treatment; the choice of the type of treatment was related to the number and site of tumors, the presence of extrahepatic disease, the liver function and the general status of the patient.

### Clinical-pathologic examination

All samples of each nodule per patient were reviewed by two pathologists (FP, PC).

We reported the size of the largest tumor nodule and the number of satellite nodules. Microscopically, we evaluated macrovascular and microvascular invasion [16], necrosis and the status of extra-tumoral parenchyma (as well as viral infection and grade of cirrhosis).

Grade of differentiation was based on Edmondson-Steiner classification [17].

An appropriate sample for the interphase molecular cytogenetic FISH study was chosen from the largest nodule of each patient, containing at least 90% of neoplastic cells.

### Fluorescence in situ hybridization analysis (FISH) molecular analysis

Interphase cytogenetic fluorescence in situ hybridization analysis was performed using commercially available locus specific identifier (LSI) MYC tri-color Break-apart Rearrangement probes, hybridizing to the band region 8q24.

The kit encounters a mixture of two probes that hybridize two opposite sides of the region located 3’ of MYC (SpectrumGreen LSI, Vysis-Abbott) and the 5’ MYC probe (SpectrumOrange LSI, Vysis-Abbott) and a centromeric (alpha-satellite DNA, SpectrumGreen) probe mapping on 8p11.1-q11.1.

Five µm sections were cut from paraffin-embedded blocks.

The procedure of FISH has been performed as detailed in previous manuscript [18]. Briefly, each probe was diluted 1:10 in tDenHyb2 buffer (Insitus, Albuquerque, NM). Ten microliters of diluted probe were applied to each slide and cover slips were placed over the slides.

Denaturation was achieved by incubating the slides at 80^°^C for 10 minutes in a humidified box; then hybridization was done at 37^°^C for 16 hours. The cover slips were then removed and the slides were immersed at room temperature in 0.5 XSSC for 2 minutes and in 2 XSSC for 2 minutes.

The slides were air dried and counterstained with 10ml DAPI/Antifade (DAPI in Fluorguard, 0.5mg/ml, Insitus, Albuquerque, NM).

The slides were examined using an Olympus BX61 (Germany) with appropriate filters for SpectrumOrange and SpectrumGreen (SpectrumOrange and SpecrumGreen LSI, Vysis-Abbott Vysis-Abbott), SpectrumAqua (centromeric probe 8, Vysis-Abbott), and the UV Filter for the DAPI nuclear counterstain (DAPI filter).

The signals were recorded with a CCD camera (Cyto-Vysion, Olympus and D-Sight/Fluo from Visia Imaging-Menarini, Florence, Italy).

Fluorescent in situ signals were evaluated on carcinomatous and normal liver adjacent parenchyma.

### Molecular analytical analysis and FISH interpretation

We initially assessed two categories: a disomic (wild) status when double fluorescent c-myc signals was observed in >90% of neoplastic nuclei, ≥3 c-myc signals in >10% of nuclei (gains of fluorescent signals).

Then, we corrected the overall fluorescent copy number signals with the copy number centromeric control probe, thus clusterized the overall results into 3 categories: a normal disomic status when observing a mean of 2 c-myc signals and 2 centromeric probe signals in >90% of nuclei; a polysomic status if we found >2 c-myc signals with 8q24/CEP8<2 in 10% of cell nuclei and a case was interpreted as “amplified” for c-myc when showing >3 c-myc signals in >10% of nuclei with 8q24/CEP8>2.

The percentages of cells harbouring the aforementioned characters were evaluated in respect to extralesional parenchyma.

### Statistical Analysis

Data were collected and analyzed with SPSS statistical software (SPSS version 19.0 Inc. Chicago Ill.) The differences between categorical and continue variables were analyzed with a chi-square test, Fisher’s exact tests and t-student test, as appropriated.

OS was defined as the time interval between treatment and death from any cause.

DFS was defined as the time interval between treatment and radiological findings of recurrence. Time was censored at the date of last follow-up assessment for patients who were still alive.

The survival curves were estimated with the Kaplan-Meier method. Clinical and pathological variables, including c-myc status were evaluated by univariate survival analysis using a log-rank test to determine any prognostic factors in patients with HCC.

Signiﬁcant variables with a p value less than 0.05 by the univariate analysis were subjected to multivariate analysis using a Cox proportional hazard regression model with backward elimination method (using a likelihood-ratio test). We reported hazard ratio and a 95% confidence interval only for the variables selected with the elimination method.

All signiﬁcance tests were 2-tailed, and a p value less than 0.05 was considered statistically signiﬁcant.

## Results

### Clinico-pathological findings

The characteristics of 65 patients included into the study are summarized in Table 1.

**Table 1:** Clinical and pathological characteristics of 65 patients affected by HCC.

Clinical-pathological features		n = 65
Gender	M	76.9%
	F	23.1%
Age		67.6 (11.7)
Cirrhosis and chronic hepatitis	Present	78.5%
	Absent	21.5%
Number of nodules		1.6 (1.0)
Size		5.48 (3.7) cm
AFP		743.5 (1814.3) ng/mL
Edmonson grading	G1	12.5%
	G2	65.6%
	G3	21.9%
Micro-vascular invasion	Present	52.3%
	Absent	47.7%
Macro-vascular invasion	Present	12.7%
	Absent	87.3%
Necrosis	Present	30.2%
	Absent	69.8%
c-myc status	Disomic	26. 1%
	Polysomic	55.4%
	Amplified	18.5%

**Figure 1:**
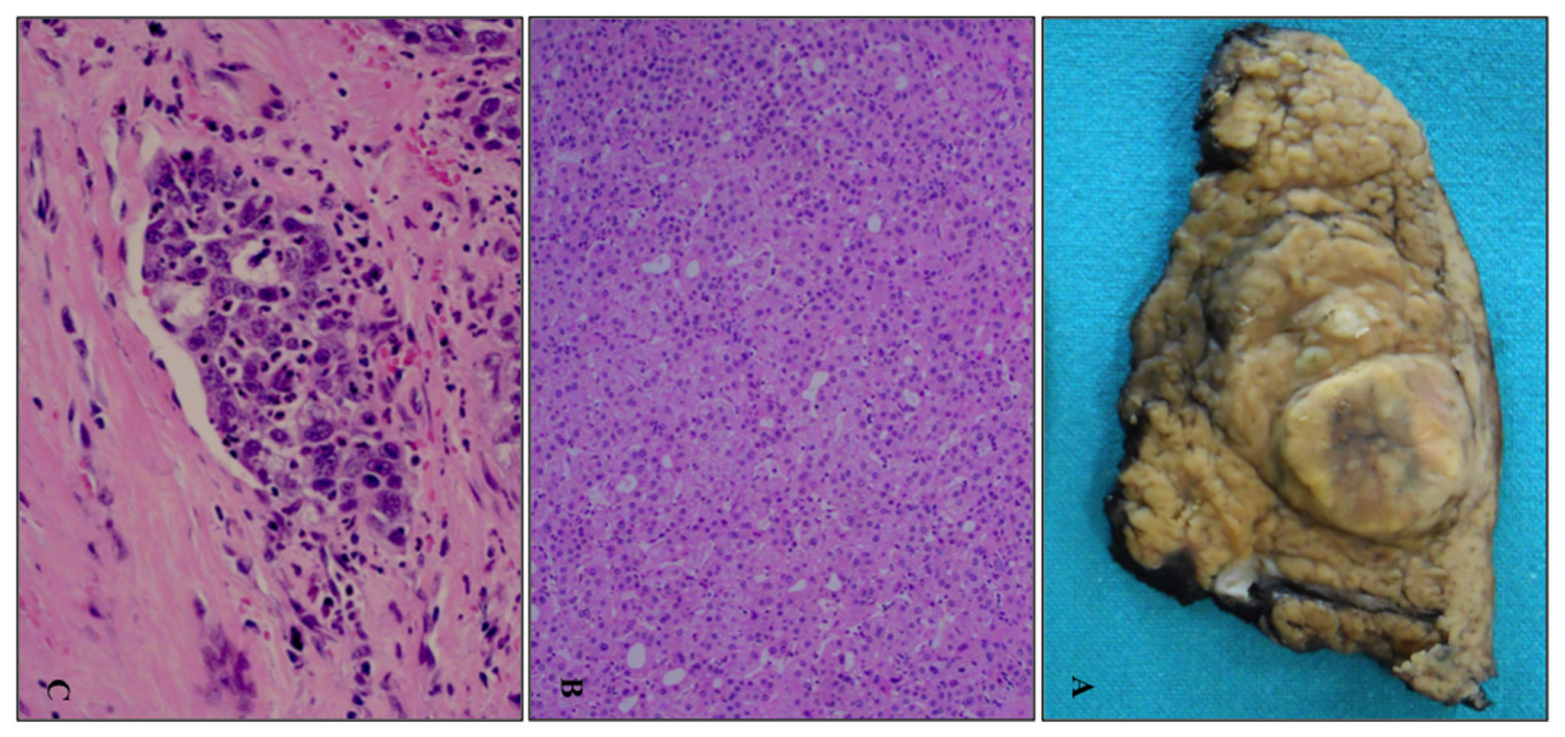
Gross and microscopic features of HCC. Surgical sample of hepatocellular carcinoma developed in a cirrhotic liver (A). Histological sample of hepatocellular carcinoma (10x, H&E) (B). Neoplastic embolus of a poorly differentiated hepatocellular carcinoma (20x, H&E) (C).

Size ranged from 1.8 to 9 (mean 5,4 cm) (Figure 1A). G1 was found in 12% (Figure 1B), G2 in 66% and G3 in 22%.

Macrovascular invasion was present in 12% of cases, whereas microvascular invasion (Figure 1C) was present in 52%. Necrosis was observed in 30% of cases.

Extralesional parenchyma was affected by chronic hepatitis (HBV in 5% and HCV in 30%) and cirrhosis in 78% of cases.

Mean follow-up was 24.6 months (SD ± 12.42 months). During follow-up, 41% of patients died with mean survival of 36.06 months (CI 95%, 31.65–40.48).

Intrahepatic recurrence developed in 56% of the 65 patients at a median time of 16 months (IC 95%, 3.55–28.45 months).

### Molecular c-myc FISH findings

From 150 to 256 (mean 179) neoplastic nuclei have been evaluated per each case.

Non-neoplastic parenchyma adjacent to tumors revealed two c-myc fluorescent signals in more than 90% of nuclei (range 90-99%, mean 94%).

**Figure 2:**
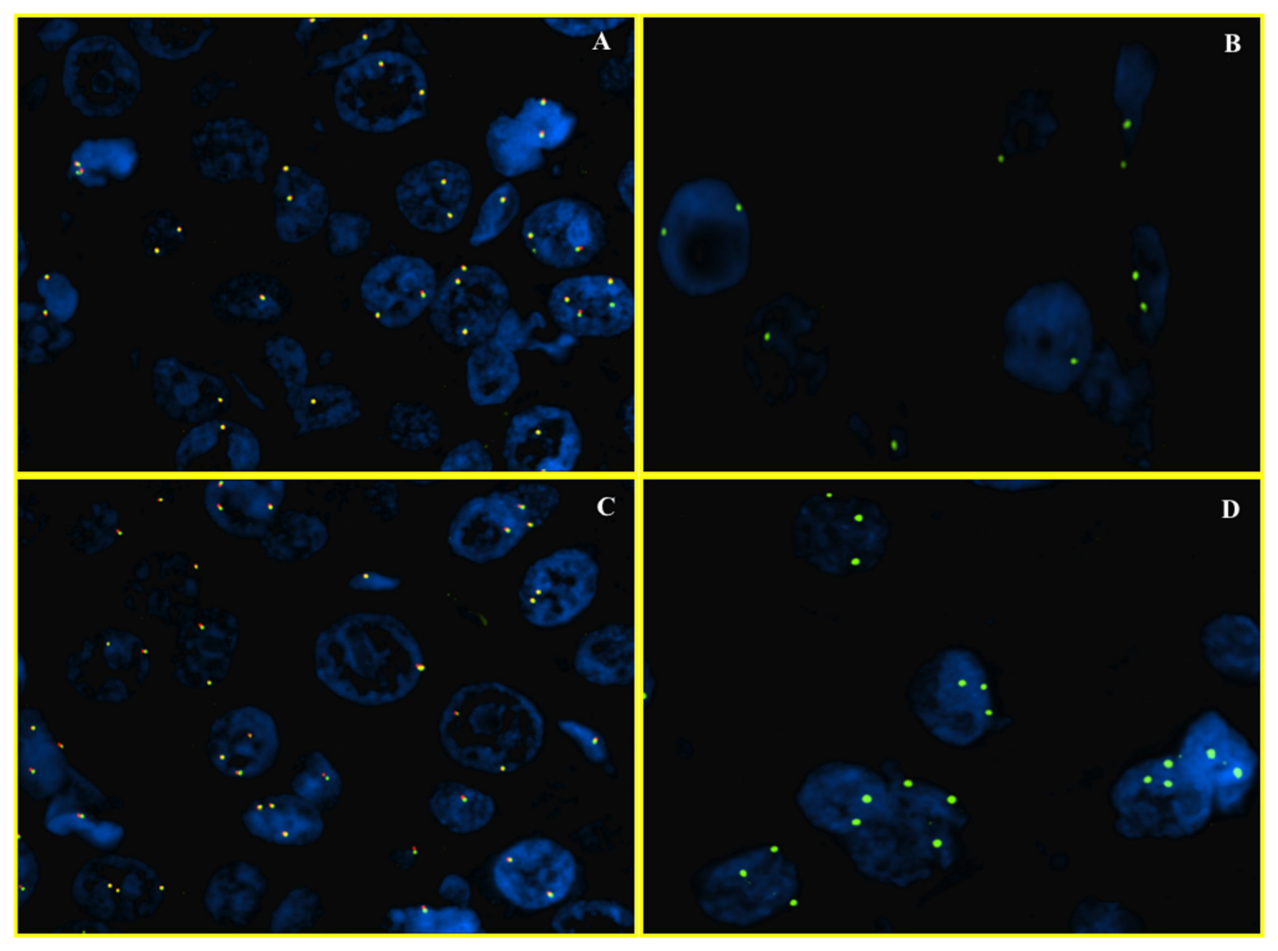
FISH analysis of c-myc (wild status and gains of 8q). Dual color fluorescence in situ hybridization with a centromere probe for chromosome 8 (CEP8, Spectrum Green), and region-specific probe for c-myc (Spectrum Orange), in representative foci of hepatocellular carcinoma. In the first two images, HCC nuclei have 2 signals both for c-myc gene (A) and for chromosome 8 (B). This case has a normal disomic status. In the other two images there are >10% of nuclei with 3 signals for c-myc (C) and for chromosome 8 (D). This case is representative of a gain for chromosome 8 (trisomy).

**Figure 3:**
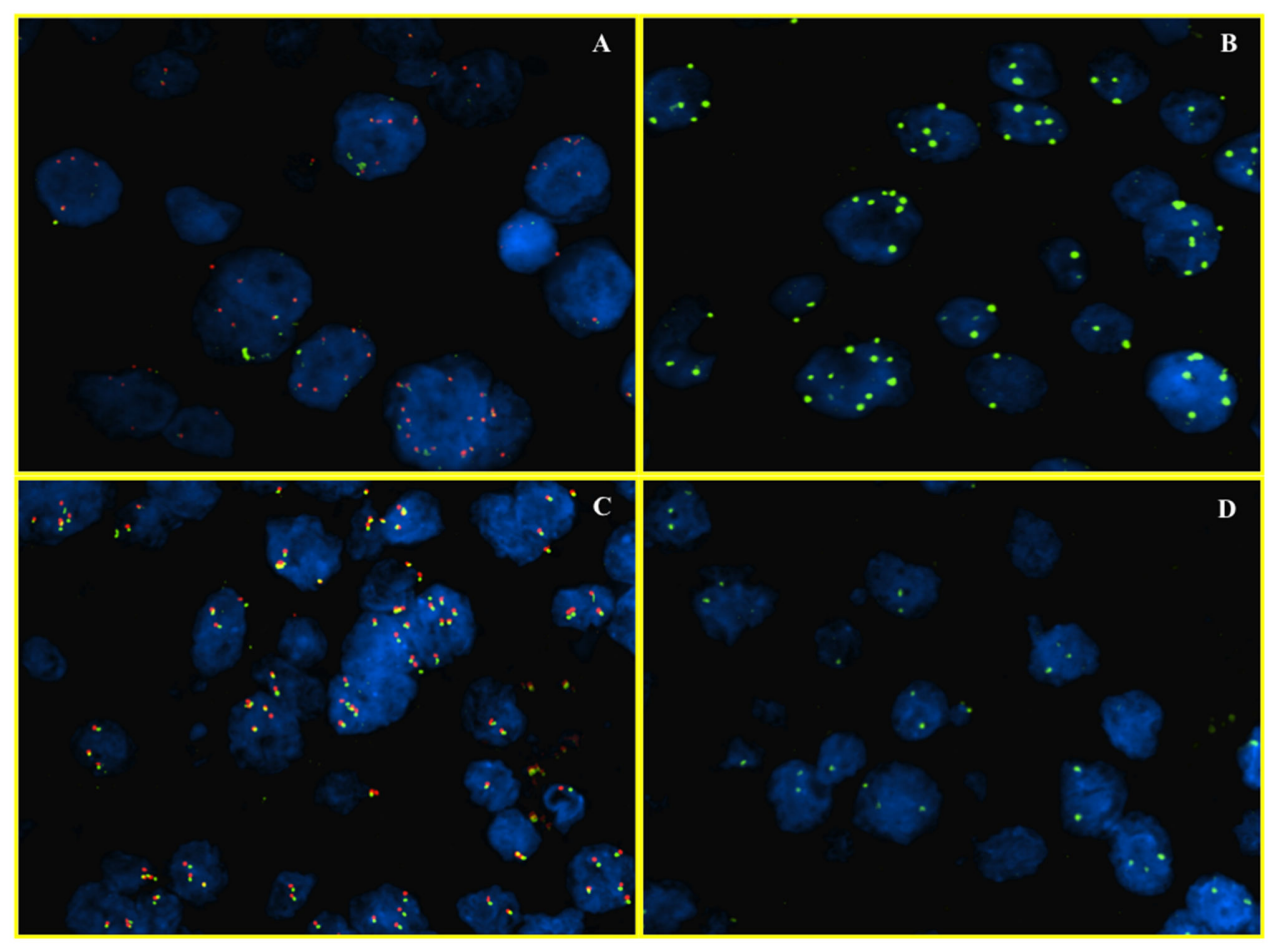
FISH analysis of c-myc (polysomy of 8q and c-myc gene amplification). Dual color fluorescence in situ hybridization with a centromere probe for chromosome 8 (CEP8, Spectrum Green), and region-specific probe for c-myc (Spectrum Orange), in representative hepatocellular carcinoma foci. In the first two images, HCC nuclei have multiple signals both for the c-myc gene (A) and chromosome 8 (B). This case has a polysomic status. In the other two images there are >10% of nuclei with multiple signals for c-myc (C) but two for chromosome 8 (D). This case is representative of amplification for c-myc.

Normal disomic status of c-myc was found in 26% of cases (17/65) (Figure 2A, 2B), while 74% (48/65) was found to be abnormal. These 48/65 cases were further subdivided in cases with ≥3 number of c-myc signals with 8q24/CEP8 < 2 (polysomic) (Figure 2C, 2D and 3A, 3B) and cases with > 3 signals of c-myc and 8q24/CEP8 ≥ 2 (true amplified) (Figure 3C, 3D).

c-myc resulted polysomic and amplified respectively in 55.4% and 18.5% of HCC.

Macrovascular invasion was statistically correlated with c-myc amplification compared to the cohorts characterized by a polysomic or disomic status (p= .003).

The other clinical (cirrhosis, dimension of tumors, number of nodules, AFP) and pathological (Edmonson-Steiner grade, microvascular invasion, tumor necrosis) prognostic factors were not significantly related with c-myc status (Table 2).

**Table 2:** c-myc status among different clinical and pathological features of 65 patients.

		Disomic	Polysomic	Amplified	P
Gender	M	47.1%	13.9%	16.7%	.037
	F	52.9%	86.1%	83.3%	
Serology	HBsAg+	11.8%	14.3%	25%	.534
	Anti-HCV Ab+	52.9%	31.4%	33.3%	
	HBsAg – and Anti-HCV ab -	35.3%	54.3%	41.7%	
Cirrhosis	Absent	29.4%	47.1%	45.5%	.505
	Present	70.6%	52.9%	54.5%	
Number of nodules	Single	70.6%	69.4%	58.3%	.820
	Multiple	29.4%	30.6%	41.7%	
Size	≤ 3 cm	47.1%	22.2%	25.0%	.202
	> 3 cm	52.9%	77.8%	75.0%	
Serum AFP	≤ 400 ng/mL	91.7%	81.8%	63.6%	.293
	> 400 ng/mL	8.3%	18.2%	36.4%	
Edmonson-Steiner grading	G1	11.8%	14.3%	8.3%	.957
	G2	64.7%	62.9%	75.0%	
	G3	23.5%	22.9%	16.7%	
Microvascular invasion	Absent	52.9%	52.8%	25.0%	.260
	Present	47.1%	47.2%	75.0%	
Macrovascular invasion	Absent	94.1%	94.3%	54.5%	.003
	Present	5.9%	5.7%	45.5%	
Tumour necrosis	Absent	82.4%	68.6%	54.5%	.291
	Present	17.6%	31.4%	45.5%	

c-myc gene amplification pattern did not vary significantly in chronically infected HCV positive patients, HBV positive patients and negative (anti-HCV Ab - and HbsAg -) patients (33.3%, 25% and 41.7%, respectively, p = 0.534).

### Overall survival (OS) univariate and multivariate analysis

At univariate analysis the presence of multiple nodules, high AFP levels (> 400 ng/ml), presence of micro- and macro-vascular invasion, tumor necrosis and amplified c-myc status were significantly associated with shorter OS (Table 3).

**Table 3:** Univariate analysis for overall survival (OS) and disease free survival (DFS).

		**OS, months (95% CI)**		**P**	**DFS, months (95% CI)**				**p**
Cirrhosis	Absent	38.65 (31.89–45.41)		.503	25.63 (18.84–32.41)				.385
	Present	35.14 (29.55–40.74)			21.74 (16.24–27.24)				
Number of nodules	Single	39.08 (34.37–43.79)		.035	27.18 (22.26–32.10)				.003
	Multiple	26.85 (19.27–34.44)			14.14 (7.45–20.82)				
Size	≤ 3 cm	41.10 (35.00–47.20)		.158	26.90 (18.61–35.19)				.335
	> 3 cm	33.82 (28.23–39.42)			22.08 (17.19–26.98)				
Serum AFP	≤ 400 ng/mL	39.48 (34.86–44.09)		.001	24.58 (19.84–29.32)				.025
	> 400 ng/mL	16.16 (9.38–22.95)			8.33 (4.37–12.29)				
Edmonson-Steiner grading	G1	33.89 (24.77–43.01)		.660	15.61 (6.28–24.94)				.438
	G2	37.35 (32.02–42.68)			23.51 (18.41–28.60)				
	G3	27.17 (20.41–33.94)			22.37 (14.98–29.74)				
Microvascular invasion	Absent	41.62 (36.53–46.71)		.011	29.87 (24.26–35.47)				.005
	Present	30.40 (23.78–37.02)			15.78 (11.34–20.22)				
Macrovascular invasion	Absent	37.25 (32.72–41.78)		.014	24.69 (20.07–29.31)				.032
	Present	18.98 (7.49–30.47)			9.22 (4.67–13.77)				
Tumour necrosis	Absent	39.87 (34.89–44.86)		.012	23.74 (18.57–28.92)				.721
	Present	26.10 (19.99–32.22)			20.56 (13.14–27.98)				
c-myc status	Not amplified	38.58 (34.06–41.11)		.018	31.60 (22.88–40.32)				.020
	Polysomy	-			21.02 (16.31–25.73)				
	Amplified	23.15 (14.11–32.19)			14.92 (4.93–24.92)				
Recurrence	≤ 12 months	28.65 (21.25–36.06)		.006	-				
	> 12 months	46.11 (41.57–50.66)							

**Figure 4:**
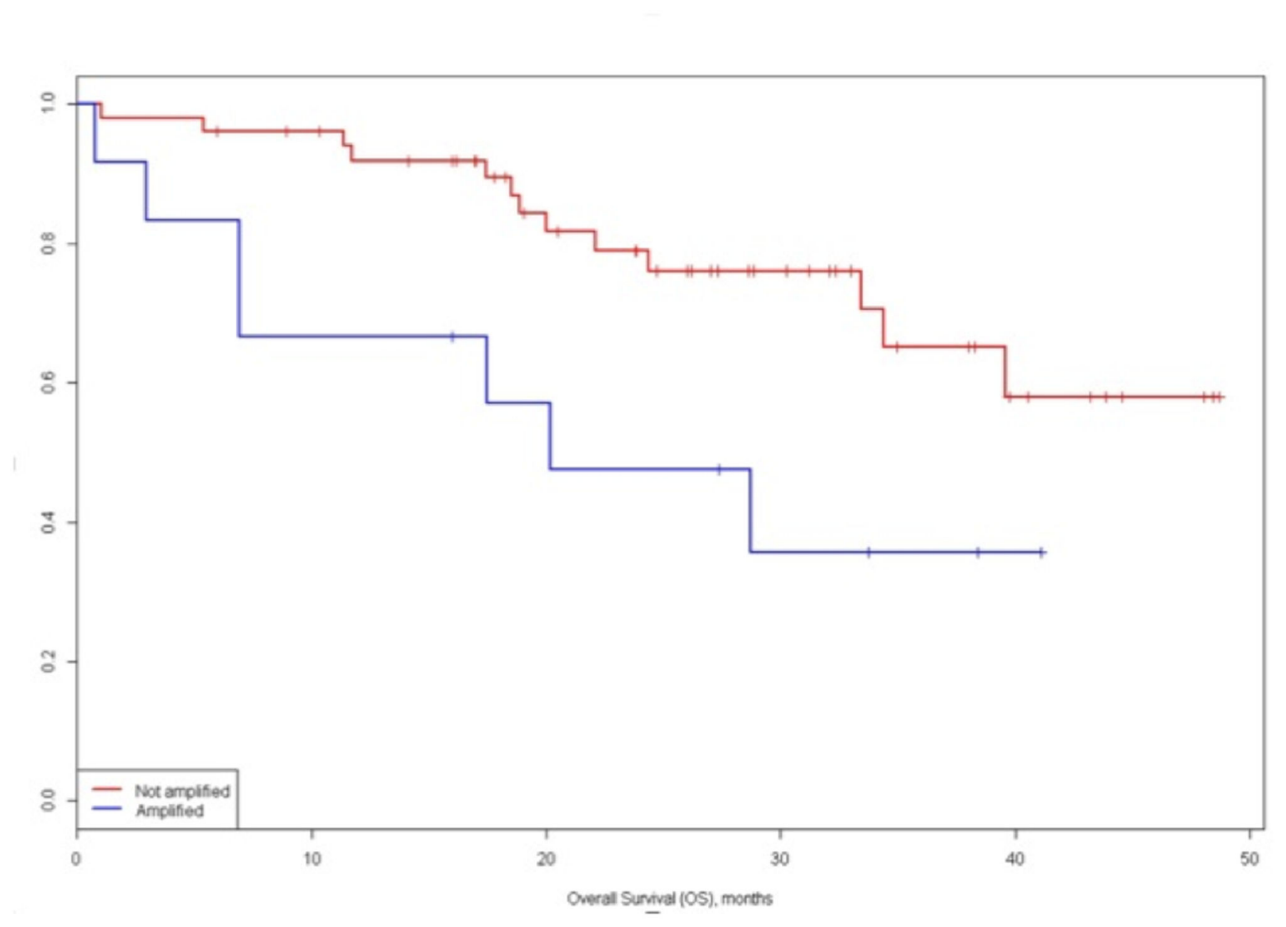
Overall survival (OS) of c-myc amplified vs. not-amplified groups.

The OS for the amplified group was lower than polysomic or disomic c-myc patterns: 3-year survival of 35.7% and 65.2% respectively (p = .018) (Figure 4). Afterwards, subgroup analysis identified a 3-year survival of 61.6% and 67.1% for disomic and polysomic groups, this difference was not statistically significant.

Multivariate analysis showed AFP and c-myc status (amplified vs. not amplified) were significant prognostic factors for OS with HRs of 1.38 (IC 95%, 1.14–1.67) and 2.82 (IC 95%, 1.02–7.79), respectively (Table 4).

**Table 4:** Multivariate analysis for overall survival (OS) and disease free survival (DFS).

		OS, HR (95% IC)	p	DFS, HR (95% IC)	P
Number of nodules	Single	-		Reference	.014
	Multiple			2.62 (1.21–5.65)	
Serum AFP	≤ 400 ng/mL	Reference	.001	Reference	.003
	> 400 ng/mL	1.38 (1.14–1.67)		1.283 (1.09–1.51)	
C-myc status	Disomic	Reference	.045	Reference	.006
	Polysomic	Reference		1.85 (.61–5.59)	
	Amplified	2.83 (1.02–7.79)		6.50 (1.84–22.98)	

### Disease free survival (DFS) univariate and multivariate analysis

**Figure 5:**
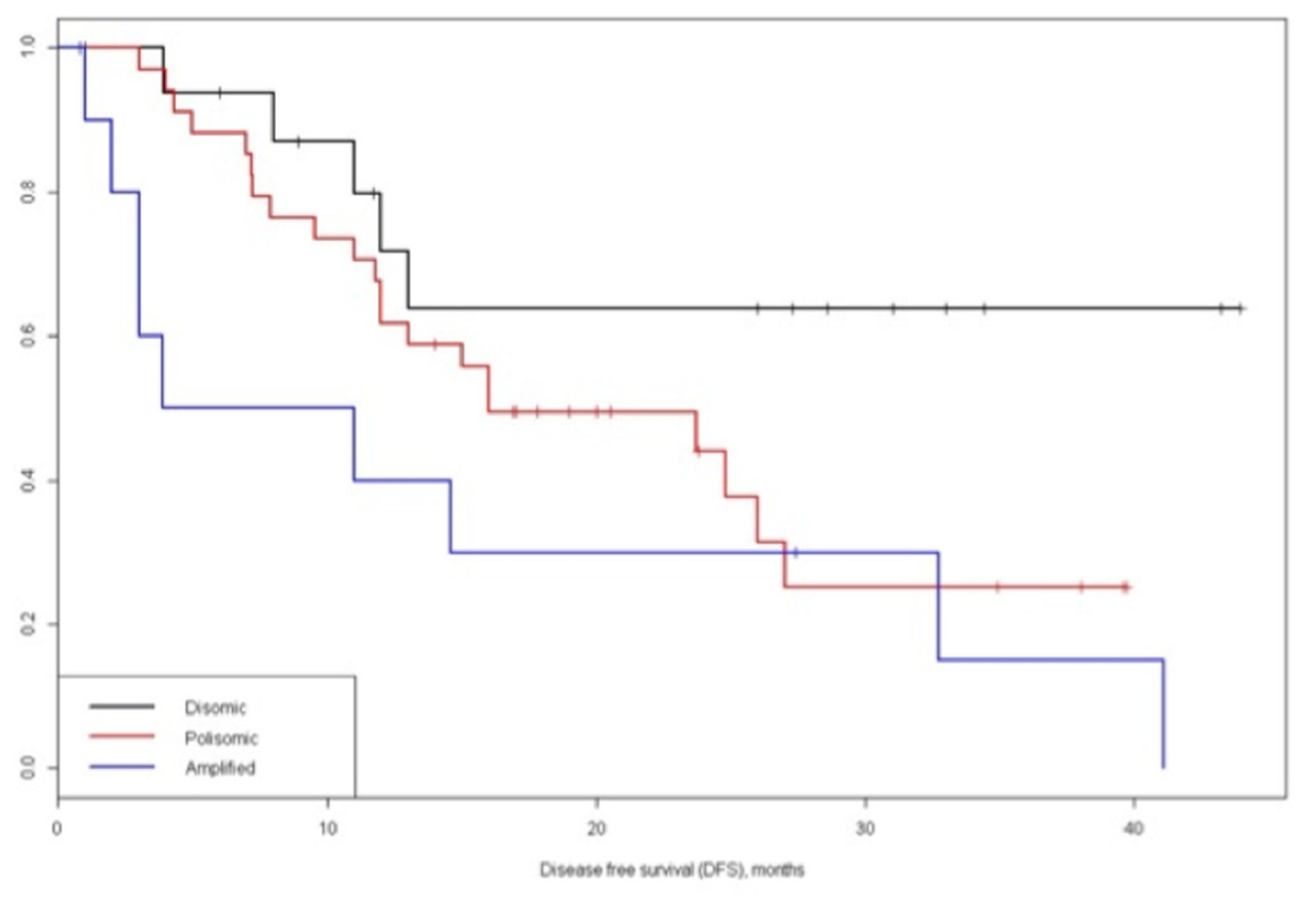
Disease free survival (DFS) of c-myc disomic, polysomic and amplified groups.

The univariate analysis for factors related with DFS identified that number of nodules (single vs. multiple), AFP levels (≤ 400 ng/mL vs. > 400 ng/mL), micro-vascular and macro-vascular invasion (absent vs. present) and c-myc status (disomic vs. polysomic vs. amplified) resulted significant prognostic factor (Table 3). Notably, the 1- and 3-year disease free survival for disomic, polysomic and amplified groups were 71.8%, 61.8%, and 30.0% and 63.8%, 25.2%, and 15.0%, respectively (P = .020) (Figure 5).

Multivariate analysis showed that the number of nodules (single vs. multifocal), AFP and c-myc status (amplified vs. disomic) were significant prognostic factors for disease free survival with HRs of 2.62 (IC 95%, 1.21–5.65), 1.28 (IC 95%, 1.09–1.51) and 6.50 (IC 95%, 1.84–22.98), respectively.

**Figure 6:**
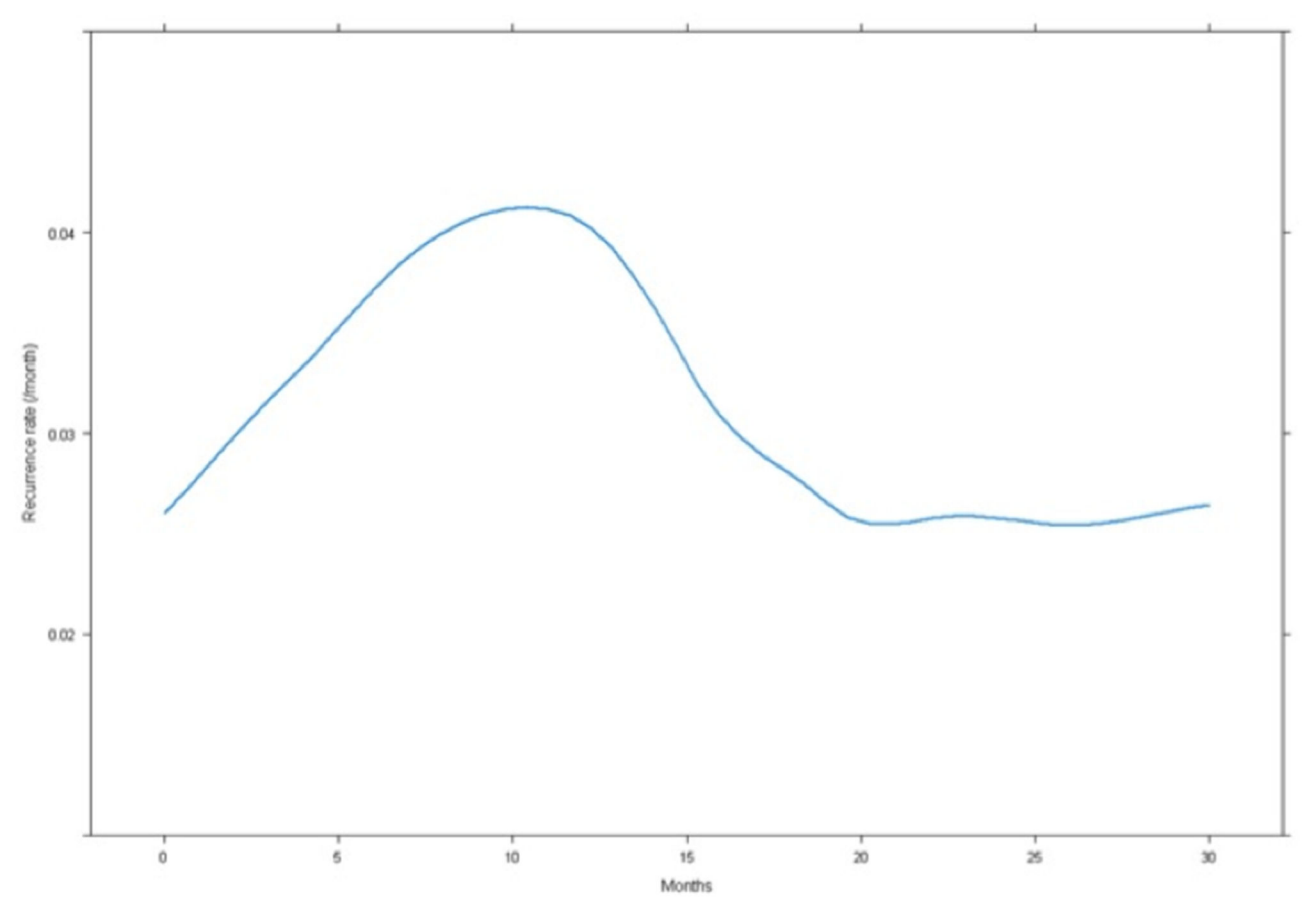
Distribution of the recurrence rate according to the time after resection, the higher rate of recurrence is achieved after 12 months.

Notably, the c-myc status was the strongest prognostic factor at multivariate analysis as show in Table 4. Figure 6 showed the hazard function of DFS estimated from our data.

Recurrence rate peaked at 12 months postoperatively (0.04/month) then gradually decrease.

## Discussion

In our study we found that: 1) c-myc is amplified in 19% of hepatocellular carcinoma at FISH molecular level, whereas show gains in 55% and set wild in 26% of respectively cases; 2) at clinical level, c-myc gene amplification is significantly correlated with DFS and OS in patients with HCC after surgical resection and identifies patients with risk of early relapse (≤12 months).

We suggest that c-myc assessment may be introduced in the clinical practice for improving prognostication (high and low risk of relapse) routinely and may have be proposed as biomarker of efficacy to anti-c-myc targeted drugs in clinical trials.

Surgical resection is one of the curative therapeutic option for HCC and it has a peri-operative mortality lower than 3% and an expected 5-year survival rates around to 50%.

The post-operative recurrence of HCC is frequent and remains one of the major causes of death. At 5 years recurrence occurs in over 70% of patients [3].

Shimada et al. [19] reported that a period of less than 12 months to recurrence was related to poor survival after recurrence. Imamura et al. [6] showed that intrahepatic HCC recurrences have an early peak of incidence (at approximately 12 months postoperatively) and recurrence rate after surgery reaches 0.4/year.

Several clinical and pathological prognostic factors have been investigated to identify patients at high risk of recurrence and poor prognosis [20,21].

Currently, none of the clinical and pathological factors can accurately estimate the risk of recurrent disease of patients undergoing surgical resection with curative intent. A promising field of research is based on molecular classification of cancer, which aims to understand the biological subclasses and optimize benefits from molecular therapies.

Actually, no molecular classification demonstrated its ability in precise prediction of survival and recurrence in clinical setting in HCC [22–26]. Among different molecular factor alteration of c-myc gene proved to be one of the important factors related with related to hepatocarcinogenesis without significant clinical correlation.

Chan et al. [27] studied c-myc in HCC applying FISH and immunohistochemistry and their data again supported the importance of c-myc in HCC carcinogenesis.

More recently, Hoshida et al. [28] have proposed a classification of HCC based on gene signature. The analysis of 603 patients indicated that there exist three major subclasses of HCC, which were defined as subclasses S1, S2, and S3. The class S2, were characterized with a poor prognosis and presented a c-myc activation signature. In addition to potential prognostication value of the c-myc molecule, targeting c-myc is becoming up-to-date because c-myc is involved in several types of other human cancer [14] and it has been discovered that some drugs, such as BET bromodomains does inhibit c-myc dependent transcription and BET family inhibitors are investigated actually for the neoplastic treatment [15].

In this study we confirmed the frequency of c-myc alteration in a subset of HCC.

Moreover, through a routinely-based FISH analysis, we identified two distinct clustered abnormalities of the gene c-myc: 55% of HCC presented with polysomy and 19% of HCC did show gene amplification.

Kawate et al. [29] studied 42 cases of HCC applying differential PCR analysis and found c-myc amplification in 33% of HCC. c-myc status was correlated with patient age, tumor size, histologic grade and a shorter disease-free survival.

Wang et al [30] studied c-myc amplification by the FISH method, comparing metastatic HCC and primary tumors. They found that c-myc was amplified in 38% of primary tumor (16 cases) and 60% of recurrent HCC. The different percentage between our study and the study of Wang et al. [30] is probably due to the different number of cases analyzed and the difference in scoring amplification. In that study in fact, Wang et al. [30] considered c-myc amplified when extra FISH signals were detected in more than 20% of tumor cells compared with control probes.

In our study we corrected the presence of fluorescent signals by the centromeric control chromosome 8. Our approach to the analytical and interpretative analysis is more aligned to the common guidelines.

Genomic heterogeneity in synchronous HCCs is an unresolved question [31–35]. Several works had demonstrated that features of the largest HCC are important prognostic factors of disease free and overall survival [36–39]. Moreover, molecular heterogeneity throughout different parts of a tumor is not clearly related with genomic predictions, eplecially in early stage HCC [40]. In our study we followed the standard practice to examine only representative region of the largest HCC when multiple lesions are identified.

Overall, the c-myc status has been found in our study be an important prognostic factor in term of DFS: the amplified c-myc status had an HR significantly higher compared to disomic and polysomic status, 6.50 (95% IC, 1.84–22.98) vs. 1.85 (95% IC, .61–5.59). Also for OS, amplified c-myc status was the strongest prognostic factors at both univariate and multivariate analysis.

Some non-clinical studies confirm that chronic liver diseases, upstream and downstream of c-myc pathways are strongly linked. Several studies conducted in vitro or in cell lines have showed that HCV and HBV hepatocarcinogenesis may involve c-myc deregulation [41–43]. To our knowledge a clear clinical relation between viral liver disease and c-myc expression has not been proven yet [27,29]. Our data showed that c-myc gene amplification patterns did not vary significantly in chronically infected HCV positive patients, HBV positive patients and negative (anti-HCV Ab - and HbsAg -) patients (33.3%, 25% and 41.7%, respectively p = 0.534).

We retrospectively investigated the c-myc status in 3 cases of hepatocellular carcinoma which had relapsed within 12 months. At c-myc status analyses we found out that two had amplification for c-myc and one was polysomic.

Our data suggest that HCC may have a unique pathway activated, which is associated with an aggressive tumor phenotype. If validated, defining HCC with gain of 8q may assist in identifying patients who could benefit for specific c-myc inhibitors or emerging agents that target the MAPK/ERK (mitogen-activated protein kinase)-c-myc related pathway [8,9].

The FISH technique is actually standardized at prognostic and predictive level in the evaluation of the Her-2/gene in breast [10] and gastric cancer [11], ALK gene in lung adenocarcinoma [12,13], 1p/19q in oligodendroglioma [13] and in several gene rearrangements in lymphoma [44].

c-myc status may be introduced in the report accompanying the macroscopic and microscopic histological diagnosis. c-myc amplification versus gains versus a disomic status and the minimum number of nuclei scored (at least 150 nuclei) may be reported. c-myc gene status on liver core biopsy in order to guide different options at surgical level for non-resectable liver cancer may be proposed.

## Conclusions

We conclude that c-myc gene amplification is an important poor prognostic factor in terms of DFS and amplification of c-myc is the strongest prognostic factors at both univariate and multivariate for overall survival. The polysomic group is numerically relevant and it will need further investigation for possible transcriptional or post-translational activation of c-myc.

The c-myc assessment may be introduced in the clinical practice both for prognostication, routinely and for efficacy to anti-c-myc targeted drugs in clinical trials.
